# Motion correction facilitates the automation of cardiac ASL perfusion imaging

**DOI:** 10.1186/1532-429X-17-S1-P51

**Published:** 2015-02-03

**Authors:** Ahsan Javed, Terrence R Jao, Krishna S Nayak

**Affiliations:** 1 Electrical Engineering, University of Southern California, Los Angeles, CA, USA; 2 Biomedical Engineering, University of Southern California, Los Angeles, CA, USA

## Background

Cardiac arterial spin labeling (ASL) perfusion imaging requires subtraction of signals in tagged and control CMR images. In recent clinical studies, perfusion reserve mapping of a single short-axis slice has required laborious manual segmentation of the LV muscle [1]. Here we demonstrate that using free open source software for automatic motion correction reduces the required manual segmentation to just 4 images (2 rest, 2 stress).

## Methods

### Framework

**Figure 1:**
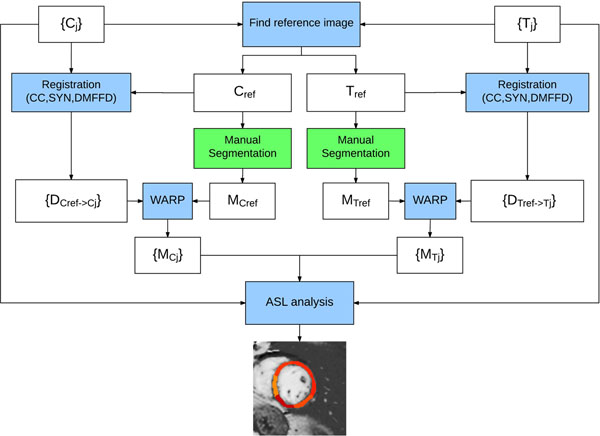
Framework to semi-automatically generate masks for LV muscle. Reference images (C_ref_, T_ref_) are automatically selected from the control {C_j_} and tagged {T_j_} images. The reference images are manually segmented to generate masks (M_Cref_, M_Tref_). Control and tagged images are then registered to their respective reference images to generate displacement fields. The reference masks are warped using the displacement fields ({D_Cref->Cj_}, {D_Tref->Tj_}) to generate masks ({M_Cj_}, {M_Tj_}) for each control and tagged image. These masks along with the control and tagged images are used for ASL analysis to generate MBF maps.

Images for rest and stress acquisition are processed similarly using the procedure in Figure 1. The control and tagged image pair that has the highest correlation to other image pairs is chosen as a "reference" (C_ref_, T_ref_), and is manually segmented to generate masks (M_Cref_, M_Tref_). Remaining control and tagged images are then registered to their respective reference images. The resulting displacement fields are applied to the reference masks to generate masks for each image. A threshold (0.8) is applied to ensure masks are binary. Myocardial blood flow (MBF) is calculated through spatio-temporal filtering, as previously described [2]. Registrations are performed using advanced normalization tools (ANTS) [3] (settings: cross correlation; symmetric diffeomorphic transform; directly manipulated free form deformation regularization).

### Validation

The proposed method was applied retrospectively to data from a published 29-patient clinical study [1]. Accuracy of masks and intra-operator variability of manual segmentation was assessed using Dice Coefficient (DC) analysis (three manual segmentations of the same datasets) and used as a benchmark for peak performance. Receiver operator characteristic (ROC) curves were generated to evaluate the proposed method and compare with manual segmentation.

## Results

**Figure 2:**
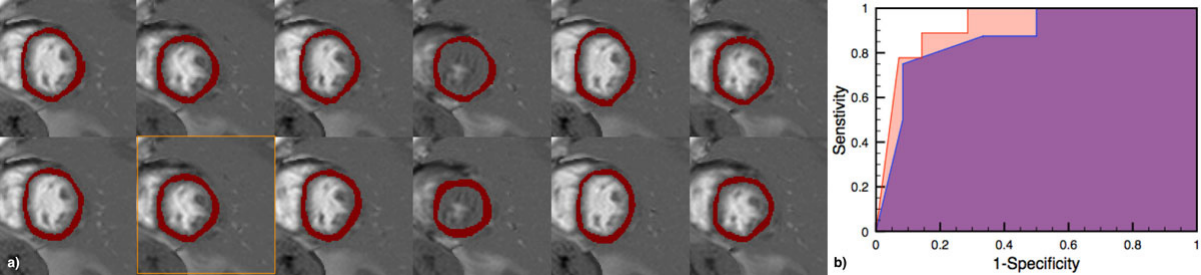
a) Masks overlaid on a series of control images. Top row shows masks from automatic segmentation and bottom row shows masks from manual segmentation. The orange box highlights the control reference frame. b) ROC curves for manual (purple) and automated (orange) approach with an AUC of 0.87 (manual) and 0.92 (automated) respectively. The higher AUC value for the automated approach is likely due to thinner masks that make it less likely to include edge pixels with partial voluming from the LV blood pool.

The intra-operator DCs were 0.75, 0.77, 0.77, and manual vs. automatic DCs were 0.70, 0.75, 0.75. This suggests that the performance of automated segmentation was comparable to the repeatability of manual segmentations. The relatively low DC values are likely due to the low resolution of the datasets; the LV region of interest is just 2-4 pixels thick. Figure 2a contains a representative example. The ROC area under the curve (AUC) for per-patient detection of angiographically significant CAD was 0.87 and 0.92 for the manual and automated approach, as shown in Figure 2b.The AUC for the automated approach shows that it has comparable diagnostic accuracy to the manual approach.

## Conclusions

This study demonstrates that motion correction reduces the need for laborious manual segmentation in myocardial ASL from 24/36 images to 4 images, and can be performed using free open source software. Motion correction improves the workflow for myocardial ASL, and may enable new experiment designs that rely on longer data collection, such as continuous monitoring of blood flow during application of stress or during intervention.

## Funding

American Heart Association; Wallace H. Coulter Foundation.
